# Multimodal neural and inflammatory correlates of neuropsychiatric symptoms across the Alzheimer’s disease spectrum

**DOI:** 10.1002/alz70861_108619

**Published:** 2025-12-23

**Authors:** Hwamee Oh, Annika Rogers, Elizabeth Kaplan, Graham Kupsaw

**Affiliations:** ^1^ Brown University, Providence, RI USA

## Abstract

**Background:**

Although genetic factors associated with the aggregation of misfolded proteins clearly play a role in Alzheimer’s disease (AD), an individual’s susceptibility to stress, often expressed as neuropsychiatric symptoms, has been shown to confer an increased risk for AD. The goal of this study was to probe a neuro‐mechanistic pathway that underlies the relationship between neuropsychiatric symptoms and AD neuropathology using multimodal neuroimaging and peripheral inflammatory markers.

**Method:**

We evaluated whether the relationship between neuropsychiatric symptoms and AD neuropathology are differentially mediated by white matter microstructural integrity, neuronal activity, and inflammatory markers along the spectrum of AD from the Alzheimer’s Disease Neuroimaging Initiative (ADNI) dataset that consists of 398 cognitively normal older adults (CNs), 817 participants with mild cognitive impairment and 307 AD patients. White matter integrity and fMRI data were processed to quantify diffusion measures and neuronal activity levels. Plasma‐based inflammatory markers were summarized using principal component analyses. Neuropsychiatric Inventory Questionnaire (NPI‐Q) scores represented individuals’ stress responses and neuropsychiatric symptoms.

**Result:**

NPI‐Q scores were significantly associated with brain Aβ indices across the entire cohort. Relationships between the neural activity, stress responses, and Aβ deposition, however, differ by diagnostic groups. As stress indicators increased, CN subjects exhibited increased activity across brain regions, while AD participants exhibited decreased activity. Moreover, as brain Aβ load increased, CN participants experienced hyperactivity, while AD participants experienced hypoactivity. White matter integrity and peripheral inflammatory markers showed diagnostic group differences, but did not significantly mediate the relationship between neuropsychiatric symptoms and AD pathologies.

**Conclusion:**

These results indicate a potential link between heightened stress responses and increased susceptibility to the development of AD pathologies through neuronal hyperactivity, especially in the early stages of AD.

